# A Hybrid Ensemble System for Time-Series Anomaly Detection in Automated Quality Control of Medical Equipment

**DOI:** 10.3390/diagnostics16131953

**Published:** 2026-06-23

**Authors:** Ziheng Zhang, Defeng Cai, Zhuo Deng, Zhicheng Du, Fuxing Zhang, Lan Ma

**Affiliations:** 1 Shenzhen International Graduate School, Tsinghua University, Shenzhen 518055, China; ziheng-z22@mails.tsinghua.edu.cn (Z.Z.); caidefeng0755@126.com (D.C.); dengz24@mails.tsinghua.edu.cn (Z.D.); duzc21@mails.tsinghua.edu.cn (Z.D.); 2 Department of Clinical Laboratory (Pathology) Centre, South China Hospital, Medical School, Shenzhen University, Shenzhen 518116, China; 3 Shenzhen YHLO Biotech Co., Ltd., Shenzhen 518116, China

**Keywords:** anomaly detection, time-series analysis, ensemble learning, clinical laboratory quality control

## Abstract

**Background/Objectives:** The accuracy and reliability of automated clinical analyzers are fundamental to patient safety and effective medical decision-making. Traditional quality control (QC) methods, which rely on periodic manual calibration and reactive maintenance, are inherently limited by high latency and labor costs; furthermore, they fail to provide continuous, real-time monitoring. This paper introduces a novel hybrid ensemble learning framework for the automated quality inspection of medical devices through the analysis of time-series reaction curves. **Methods:** Our system integrates three heterogeneous anomaly detection paradigms: an Enhanced Dynamic Time Warping (DTW) detector for robust non-linear pattern matching, a Shape Template Matching (STM) detector that mimics expert clinical logic by analyzing morphological features in a normalized shape space, and a specialized Time-series Variational Autoencoder (TimeVAE) for deep representation learning. The outputs of these detectors are fused using a weighted ensemble strategy, which is specifically designed to prioritize the minimization of false negatives—a critical requirement in medical diagnostics. **Results:** We evaluate our framework on a comprehensive, multi-center real-world dataset comprising seven distinct biochemical assays. Experimental results demonstrate that our proposed method achieves superior performance, attaining a 0% false negative rate on CRE and DBIL assays and outperforming all baseline methods on the other five datasets. An ablation study confirms the model’s robustness even with limited training data, and a comparative analysis against eight state-of-the-art baseline methods further validates the effectiveness of our domain-optimized ensemble approach. **Conclusions:** The system provides a robust, interpretable, and highly automated solution for transitioning from reactive maintenance to proactive, real-time quality assurance in clinical laboratories.

## 1. Introduction

In modern clinical practice, automated biochemical analyzers serve as the cornerstone of laboratory medicine, as their outputs directly influence patient diagnoses and treatment plans. The reaction curves generated during chemical assays encapsulate critical kinetic information; any deviation caused by hardware failure, reagent degradation, or pipetting errors can lead to erroneous diagnostic reports, potentially compromising patient safety. Conventionally, quality assurance for such equipment relies on periodic manual calibrations, scheduled maintenance, and reactive repairs following a detected failure. This paradigm suffers from significant limitations, including response latency, high labor costs, and an inability to perform continuous, real time monitoring [1,2]. An undetected malfunction poses a direct risk to patient safety and undermines the quality of medical care. To address these challenges, this research develops an automated quality inspection system. By applying intelligent analysis to the multi-dimensional data generated during device operation—including raw sensor outputs and reaction curves—the system enables real time status monitoring, proactive anomaly warning, and preliminary fault attribution, with the overarching goal of enhancing the reliability of medical devices and the trustworthiness of their diagnostic results.

Traditional QC methods, such as Westgard rules and manual review, are inherently reactive and often fail to capture intermittent or early stage micro anomalies. Existing industrial anomaly detection algorithms, including simple Statistical Process Control (SPC), struggle with the inherent noise and biological variability of medical data. A significant challenge is that absolute signal amplitudes in medical assays are often not the sole indicator of abnormality; instead, the morphological pattern of the reaction curve—the “shape” over time—carries critical diagnostic information that conventional methods frequently overlook. Time series anomaly detection is a well established field [3,4]. Traditional statistical methods, such as ARIMA, often fail when confronted with non-stationary or complex medical data [5,6,7]. Simpler machine learning models, including Isolation Forest (IF) and One Class SVM (OCSVM), have been applied for their efficiency but struggle to capture the intricate morphological characteristics of reaction curves, leading to suboptimal performance [8,9,10,11].

Distance-based methods, particularly Dynamic Time Warping (DTW), are popular for comparing time series. However, standard DTW can be sensitive to noise and lacks a mechanism to handle natural variations in normal patterns. With the advent of deep learning, reconstruction based models have become prominent [12,13,14]. Architectures like the Vanilla Autoencoder (AE) and LSTM Autoencoder [15] learn to compress normal data but may not be optimized for the specific characteristics of medical curves [16,17,18,19,20]. More advanced models, such as OmniAnomaly, leverage variational autoencoders (VAEs) and stochastic recurrent networks to model complex temporal dependencies [21,22]. Other approaches, such as those based on Generative Adversarial Networks (GANs), have also been explored for identifying outliers [23,24]. The Matrix Profile (MP) offers interpretability by identifying anomalous subsequences but focuses on local discord rather than the holistic integrity of an entire reaction curve [25]. Existing methods, while powerful in their respective domains, often fall short when applied to medical reaction curves [26,27]. They frequently lack domain specific optimization [28], struggle to balance sensitivity and specificity, and offer insufficient interpretability for clinical review [21,29,30]. This motivates the development of a hybrid ensemble framework tailored to these unique challenges.

In this paper, we propose a novel hybrid ensemble learning framework for the automated quality inspection of medical devices through the analysis of time series reaction curves. Our contributions are four-fold. First, we introduce a domain optimized ensemble framework that synergistically combines three disparate anomaly detection paradigms: distance based matching (Enhanced DTW), morphological feature engineering (Shape Template Matching), and deep representation learning (TimeVAE). This multi-faceted approach ensures robustness by capturing a wide spectrum of anomaly types. Second, we present a biomimetic Shape Template Matching detector that employs Z score normalization to isolate a “shape space” from absolute intensity, mimicking the intuition of clinical experts who prioritize morphological trends. This significantly enhances robustness against gain variations and provides high interpretability through direct visual comparison. Third, our system is designed for high automation, with a standardized preprocessing pipeline and multi-view statistical feature extraction that enables end-to-end processing of raw data without manual intervention, and all hyperparameters are centralized for flexible configuration. Fourth, through a balanced loss function in the TimeVAE and a carefully designed ensemble strategy, our system achieves 0% false negative rate on two well-characterized assays (CRE, DBIL) and maintains near-zero FNR on all other test datasets, meeting the stringent safety requirements of clinical diagnostics.

## 2. Materials and Methods

### 2.1. Overall Framework

**Figure 1:**
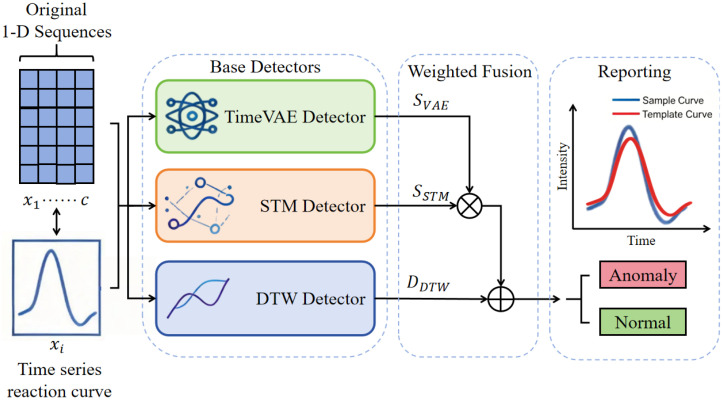
Overall framework of the automated Anomaly Detection System.

The proposed Ensemble Anomaly Detection (Ensemble-AD) system is an end-to-end automated quality inspection framework designed for time-series reaction curves generated by clinical biochemical analyzers and the overall framework of the proposed system is illustrated in Figure 1. The pipeline consists of four sequential stages: data input and standardization, parallel anomaly detection, weighted score fusion, and final decision-making. Specifically, each input curve is simultaneously fed into three complementary detectors: an Enhanced Dynamic Time Warping (DTW) detector, a Shape Template Matching (STM) detector, and a Time-Series Variational Autoencoder (TimeVAE) detector. The outputs of these heterogeneous modules are integrated via a carefully designed cascade fusion strategy to produce a robust, clinically reliable classification result.

### 2.2. Base Detectors

#### 2.2.1. Detector 1: Enhanced DTW Detector

The Enhanced DTW detector leverages multi-template pattern matching to capture global morphological anomalies while tolerating physiological and instrumental variations in normal reaction curves. First, a comprehensive set of statistical, dynamic, and morphological features is extracted to characterize each time-series sample. K-means clustering is then applied to the feature vectors of normal training samples, and the cluster centroids serve as representative normal templates. For a given test sample, a composite similarity score is computed by combining Pearson correlation, dynamic time warping distance, and Euclidean distance, weighted to emphasize global shape consistency. The sample is flagged as anomalous if its maximum similarity score across all templates falls below a predefined threshold.

- Feature Extraction: Statistical, dynamic, and morphological features are extracted and combined to form feature vector for each curve.
- Template Generation: K-Means clustering is applied to the feature vectors of normal training samples. The centroid of each resulting cluster serves as a “normal” template, representing a distinct operational mode.
- Similarity Calculation: For a test sample *y*, a composite similarity score *S* is computed against each template *t*:
  $$S\left(y,t\right)=\omega_{1}\times Corr\left(y,t\right)+\frac{\omega_{2}}{1+\mathrm{DTW}(y,t)}+\frac{\omega_{3}}{\left(1+||y-t|{|}^{2}\right)}$$
  where Corr is the Pearson correlation coefficient, DTW is the DTW distance, and $||y-{T||}^{2}$ is the Euclidean distance.
- Anomaly Detection: The sample is compared against all templates, and the maximum similarity score is identified. If this score falls below a predefined fixed threshold, the curve is flagged as anomalous.

#### 2.2.2. Detector 2: Shape Template Matching (STM) Detector

The Shape Template Matching detector is engineered to prioritize curve morphology over absolute signal amplitude, thereby achieving robustness against baseline shifts and gain variations. During training, all normal curves are normalized into a shape-invariant space using Z-score standardization, eliminating intensity and offset information. A unified shape template is constructed by averaging the normalized normal curves. The anomaly threshold is determined based on the distribution of distances between training samples and the template, ensuring adaptability to inherent within-class variations. During inference, a test curve is normalized using its own mean and standard deviation, and its distance to the shape template is used to judge abnormality.

Training Phase:

1. Normalization into Shape Space: Given a set of normal curves ${\left\{x_{i}\right\}}_{i=1}^{N}$, where $x_{i}\in R^{L}$, each curve is Z-score normalized to have zero mean and unit variance. This transformation isolates the shape by removing amplitude and offset information:
  $${\tilde{x}}_{i}=\frac{x_{i}-\mu_{i}}{\sigma_{i}+\epsilon }$$
  where $\mu_{i}$ and $\sigma_{i}$ are the mean and standard deviation of $x_{i}$, and ϵ is a small constant.
2. Template Creation: A single shape template *t* is computed by averaging all normalized curves: $t=\frac{1}{N}\sum_{i=1}^{N}{\tilde{x}}_{i}$.
3. Threshold Determination: The Euclidean distance $d_{i}=\left|\right|{\tilde{x}}_{i}-t{\left|\right|}^{2}$ is calculated for each training sample. The anomaly threshold τ is set based on the distribution of these distances, typically as a scaled percentile to provide a margin for natural variation:
  $$\tau =P_{95}\left(\left\{d_{i}\right\}\right)\times \alpha$$
  where $P_{95}$ is the 95th percentile and α is a margin coefficient.

Detection Phase:

A test curve *y* is normalized to $\tilde{y}$ using its own mean and standard deviation. Its distance to the template, $d_{dest}=\left|\right|\tilde{y}-t{\left|\right|}^{2}$, is calculated. If $d_{dest}>\tau$, the curve is declared anomalous.

#### 2.2.3. Detector 3: TimeVAE Detector

The TimeVAE detector is a dedicated deep representation learning model tailored for the non-linear, kinetic characteristics of medical reaction curves. It comprises a sequence encoder, a statistical feature encoder, a latent fusion module, and a reconstruction decoder. The sequence encoder uses one-dimensional convolutional layers and a self-attention mechanism to capture local and temporal dependencies. The statistical feature encoder encodes high-level kinetic properties such as mean, variance, skewness, and kurtosis through a multi-layer perceptron [17,31,32]. The two latent representations are concatenated and fed into the decoder to reconstruct the original time series. Anomalies are identified based on reconstruction error and latent distribution divergence [15,33].

**Figure 2:**
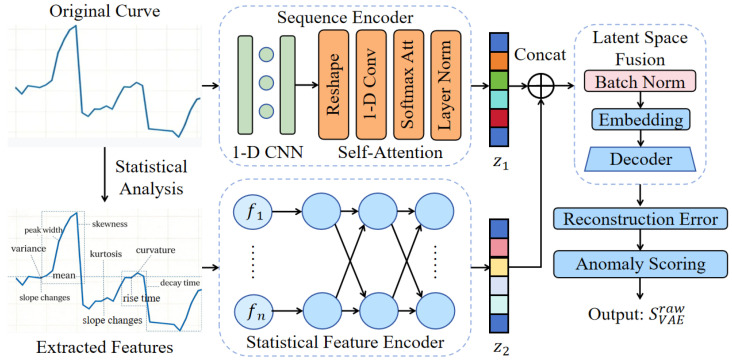
Architecture of the TimeVAE model.

As shown in Figure 2, the model architecture includes:

- Sequence Encoder: A series of 1-D convolutional layers capture local features, followed by a self-attention mechanism to weigh the importance of different time steps, and finally global average pooling to produce a fixed-size representation of the curve’s latent mean and variance [34].
- Statistical Feature Encoder: A parallel MLP encodes statistical features such as mean, variance, skewness, kurtosis extracted from the raw curve.
- Latent Space Fusion: The latent variable $z_{1}$, sampled from the sequence encoder’s output, is concatenated with the encoded statistical features $z_{2}$.
- Decoder: A series of transposed convolutional layers reconstruct the original time series from the fused latent representation.

### 2.3. Ensemble Detector: The Fusion Strategy

The Ensemble Detector integrates the outputs of the three individual modules to make a final decision [35,36]. We selected a weighted calculation approach for its superior performance in achieving near-zero false negatives [19]. The fusion procedure, including score normalization, is formalized as a stepwise decision algorithm (Algorithm 1). Let $S_{\mathrm{STM}}^{\mathrm{raw}}\left(x\right)$ and $S_{\mathrm{VAE}}^{\mathrm{raw}}\left(x\right)$ denote the raw anomaly scores produced by the Shape Template Matching detector and the TimeVAE detector, respectively, for a test sample *x*. These raw scores are first normalized to [0,1] using min-max scaling based on their empirical distributions observed on a validation set V. For a detector d∈{STM,VAE}, the normalized score is computed as:

$$S_{d}\left(x\right)=\frac{S_{d}^{\mathrm{raw}}\left(x\right)-\min_{v\in V}S_{d}^{\mathrm{raw}}\left(v\right)}{\max_{v\in V}S_{d}^{\mathrm{raw}}\left(v\right)-\min_{v\in V}S_{d}^{\mathrm{raw}}\left(v\right)+\epsilon },$$

where ϵ is a small constant. The Enhanced DTW detector directly outputs a binary decision $D_{\mathrm{DTW}}\left(x\right)\in \left\{0,1\right\}$, with 1 indicating an anomaly, and 0 for normal.
**Algorithm 1** Ensemble fusion strategy with score normalization**Require:** 1:•        Test sample *x*•        Raw Shape Template Matching score: $S_{\mathrm{STM}}^{\mathrm{raw}}\left(x\right)\in R$•        Raw TimeVAE anomaly score: $S_{\mathrm{VAE}}^{\mathrm{raw}}\left(x\right)\in R$•        Binary output from Enhanced DTW: $D_{\mathrm{DTW}}\left(x\right)\in \left\{0,1\right\}$•        Validation set V (for min-max bounds)•        Secondary fusion threshold: $\tau_{\mathrm{fused}}$**Ensure:** Final binary decision $D_{\mathrm{final}}\left(x\right)\in \left\{0,1\right\}$, where 1 denotes anomalous and 0 normal. 2:                     ▹ Step 0: Normalize raw scores to [0,1]3:$S_{\mathrm{STM}}\leftarrow \mathrm{Normalized}\;S_{\mathrm{STM}}^{\mathrm{raw}}\left(x\right)$4:$S_{\mathrm{VAE}}\leftarrow \mathrm{Normalized}\;S_{\mathrm{VAE}}^{\mathrm{raw}}\left(x\right)$5:                      ▹ Step 1: Preserve all DTW positives6:**if** $D_{\mathrm{DTW}}\left(x\right)=1$ **then**7:     $D_{\mathrm{final}}\leftarrow 1$8:**else**                ▹ Step 2: Fuse deep learning and shape scores9:     $S_{\mathrm{fused}}\leftarrow S_{\mathrm{STM}}\times S_{\mathrm{VAE}}$             ▹ Step 3: Apply secondary threshold10:      **if** $S_{\mathrm{fused}}>\tau_{\mathrm{fused}}$ **then**11:         $D_{\mathrm{final}}\leftarrow 1$12:      **else**13:         $D_{\mathrm{final}}\leftarrow 0$14:      **end if**15:**end if**16:**return** $D_{\mathrm{final}}$

The entire decision logic can be equivalently expressed as a compact rule:

$$D_{\mathrm{final}}=D_{\mathrm{DTW}}\;\vee \;\left(S_{\mathrm{STM}}\times S_{\mathrm{VAE}}>\tau_{\mathrm{fused}}\right),$$

where $D_{\mathrm{DTW}}\in \left\{0,1\right\}$ and the inequality evaluates to 1 if true and 0 otherwise.

This cascaded rule explicitly prioritizes the detection capability of DTW, ensuring that any strong morphological mismatch identified by the distance-based detector will not be overridden by other modules, which prioritizes minimizing false negatives—critical for patient safety while balancing precision.

This design offers three key advantages. First, any sample flagged by the Enhanced DTW detector is immediately classified as anomalous, ensuring that no strong distance-based morphological mismatch is overridden by other modules—a critical property for obtaining satisfying false negatives rate. Second, multiplicative fusion $S_{\mathrm{STM}}\times S_{\mathrm{VAE}}$ yields a high value only when both detectors agree on a high anomaly probability; if either detector is uncertain, the product remains low, effectively acting as a logical AND in the continuous domain and thereby reducing false positives. Third, the min-max normalization step renders the scores from heterogeneous detectors commensurate, enabling principled fusion without arbitrary scaling. All thresholds are configurable and were determined via grid search on the validation set to maximize F1 while maintaining zero false negatives on the training distribution.

## 3. Results

### 3.1. Data Source and Availability

The raw experimental time-series data utilized in this research originated from the SHENZHEN YHLO BIOTECH Co., Ltd. (Shenzhen, China). The data were collected during the initial installation and calibration phase of the analyzer platforms at multiple clinical institutions. During this commissioning process, standardized quality control materials with known analyte concentrations were repeatedly measured to verify instrument performance. Each resulting reaction curve was independently reviewed by equipment engineers and clinical laboratory experts, who jointly assigned a binary label (normal or anomalous) based on their domain knowledge of the expected kinetic behavior of each assay. A curve was flagged as anomalous if its morphological pattern deviated from the well-characterized reference profile of the standard material, as determined by expert consensus. In addition to the binary label, the annotators recorded the suspected root cause of the anomaly (e.g., pipetting errors, reagent degradation, or optical path obstruction), which provides a foundation for future fine-grained fault classification. All annotations were cross-validated by at least two experts to ensure consistency. Prior to analysis and inclusion in this manuscript, all datasets underwent a rigorous, multi-stage anonymization and de-identification protocol. This process ensured the complete removal or masking of any direct or indirect identifiers that could link the data back to specific individuals, instruments, or proprietary operational contexts outside the scope of aggregated signal analysis. To promote transparency and facilitate scientific reproducibility, the processed and analyzed experimental datasets underpinning the results presented in this document are available for verification. While the original, raw experimental data remains securely archived in compliance with data retention policies, it can be made available to qualified researchers upon reasonable request. Requests for access to the original, raw experimental data must be directed in writing to the Corresponding Author of this manuscript for formal review and approval.

### 3.2. Data Preprocessing and Statistical Feature Extraction

To facilitate robust anomaly detection across heterogeneous detectors, we implement a sequential preprocessing pipeline that transforms raw reaction curve data into standardized inputs and statistical features. The preprocessing stage normalizes signal variations and extracts morphological characteristics that are informative for subsequent anomaly scoring. We consider following statistical features:

- Basic statistical features: mean, variance, skewness, kurtosis, minimum/maximum value and their corresponding positions;
- Dynamic features: integral of the curve, average and variance of first-order and second-order derivatives, maximum slope and rise/decay rates;
- Morphological features: peak width at half maximum, number of local extrema, overall curve length and curvature.

These features are fundamental for template generation in the Enhanced DTW detector and work as additional input to the TimeVAE detector. This multi-view feature extraction enriches the model with domain knowledge about reaction kinetics, improving the robustness of anomaly detection.

### 3.3. Experimental Setup

The data were collected from multiple clinical centers and consist of real-world reaction curves from automated biochemical analyzers, covering seven biochemical assays: ALP, AST, CRE, DBIL, GGT, TBIL, and TP. Experiments were conducted on a server with an NVIDIA RTX 3090 GPU, CUDA 12.9. Baseline methods, including Isolation Forest [6], One-Class SVM [7], Global DTW [37], Vanilla AE [16], LSTM-AE [15], Matrix Profile [25], GAN-based detector [23], and OmniAnomaly [38], were implemented using Scikit-learn [39] and PyTorch 2.8. Training employed the Adam optimizer (learning rate $10^{-3}$, batch size 64), early stopping with patience 20 epochs, and a maximum of 200 epochs [40,41]. The anomaly score for a test sample was computed as the min-max normalized sum of the mean squared reconstruction error and KL divergence, using statistics derived from the validation set. Model performance was evaluated using 5-fold cross-validation, with each fold preserving the original class distribution. Hyperparameters for each detector were determined via grid search on a validation set (10% of training data) and fixed before final evaluation.

### 3.4. Comparison with State-of-the-Art Methods

We compared the proposed Ensemble-AD against eight baseline methods across all seven datasets. Table 1 reports the F1-scores, while Table 2 presents the false negative rates (FNR, %).

We compared the proposed Ensemble-AD against eight baseline methods across all seven datasets. Table 1 reports the F1-scores, and Table 2 presents the false negative rates (FNR, %). The baselines include traditional machine learning methods (Isolation Forest [6], One-Class SVM [7]), distance-based matching (Global DTW [37]), generic deep learning models (Vanilla AE [16], LSTM-AE [15]), the interpretable Matrix Profile [25], and advanced deep anomaly detectors (GAN-based [23], OmniAnomaly [38]).

Ensemble-AD consistently achieves the highest or near-highest F1-score on every dataset and is the only method that attains a false negative rate of 0% on CRE and DBIL assays. This perfect FNR is a direct consequence of our design philosophy, which prioritizes the minimization of false negatives over false positives. In clinical quality control, a missed anomaly (false negative) can lead to undetected equipment malfunction, potentially resulting in erroneous patient results and compromised safety. Conversely, a false positive triggers a manual inspection, which carries a manageable cost. Therefore, we intentionally engineered the ensemble fusion strategy and the loss functions of the TimeVAE detector to strongly penalize false negatives. The results confirm that this clinically driven trade-off has been successfully achieved. On the CRE and DBIL assays, which exhibit relatively well-defined normal patterns, Ensemble-AD improves the F1-score by 3.4% and 4.3% over the second-best method (OmniAnomaly), respectively.

Traditional methods (Isolation Forest, One-Class SVM) perform poorly on all datasets, with F1-scores typically below 0.66. This confirms their inability to capture the complex temporal and morphological patterns of reaction curves. Distance-based Global DTW improves upon these but still yields FNRs of 6–7%, validating the need for our enhanced multi-template DTW approach. Generic deep learning models (Vanilla AE, LSTM-AE) achieve competitive F1-scores on some datasets, yet they fail to reduce FNR to an acceptable level for clinical deployment. Even the strongest baseline, OmniAnomaly, exhibits FNRs of 2.2–5.6% across the five challenging assays, meaning that dozens of true anomalies would be missed in a large-scale deployment. In contrast, Ensemble-AD reduces false negatives greatly, demonstrating the effectiveness of our hybrid fusion strategy and domain-specific design choices.

For a clinical laboratory processing thousands of reaction curves daily, unacceptable FNR poses a potential risk to patient safety [42,43,44]. Our system therefore provides a level of reliability that aligns with the stringent requirements of medical quality assurance.

It is important to recognize that the seven biochemical assays studied in this work exhibit substantially different degrees of class imbalance. The proportion of anomalous samples in the test sets ranges from 0.6% (TP, with approximately one anomaly per cross-validation fold) to 18.7% (CRE, with 33 anomalies per fold). In highly imbalanced settings, the F1-score—while informative—can present an incomplete picture of model performance, as it is influenced by the positive class prevalence and does not account for true negatives. To provide a more comprehensive and clinically meaningful evaluation, we report two more metrics that are specifically recommended for imbalanced classification: the Area Under the Precision–Recall Curve (AUPRC) and the Matthews Correlation Coefficient (MCC). AUPRC measures the trade-off between precision and recall across all possible decision thresholds; its chance-level baseline equals the anomaly rate of the dataset, making it particularly informative for comparing methods across datasets with different class priors. MCC summarizes the complete confusion matrix into a single correlation coefficient which is widely regarded as a balanced metric that fairly reflects performance on both the minority and majority classes. The AUPRC and MCC results are reported in Table 3 and Table 4 respectively.

The AUPRC and MCC results are largely consistent with the F1-score and FNR trends reported in Table 1 and Table 2. On the CRE and DBIL assays, Ensemble-AD achieves the highest AUPRC (0.872 and 0.758, respectively) and the highest MCC (0.885 and 0.785), confirming the effectiveness of the hybrid ensemble strategy on these well-characterized assays. On the remaining five datasets, Ensemble-AD is competitive with or outperforms the best baseline methods. Notably, GAN-based and LSTM-AE each achieve the best AUPRC and MCC on the highly imbalanced ALP and AST datasets (anomaly rate 0.7%), indicating that purely data-driven deep models can also yield strong ranking performance on extremely scarce anomaly data. The MCC values further corroborate that our ensemble approach maintains a balanced trade-off between sensitivity and specificity across all datasets, with MCC ranging from 0.572 (AST) to 0.885 (CRE). The lower absolute AUPRC values on the highly imbalanced datasets (ALP, AST, TBIL, TP) are a direct consequence of the small number of anomalous samples available for evaluation—each cross-validation fold contains only one or two anomalies—and should not be interpreted as poor detection capability.

To assess model robustness, we varied the number of training samples for each dataset, using proportions of the full training set (10%, 20%, 30%, 50%, and full).

Table 5 reports the F1-scores. The results demonstrate remarkable robustness and powerful few-shot ability of our ensemble framework. This indicates that the hybrid framework strongly reduces the dependency on large-scale labeled datasets, which is especially valuable in clinical environments where annotating anomalies is costly and time-consuming.

### 3.5. Ablation Study

We performed two sets of ablation experiments to quantify the contribution of each base detector and to validate the design of our ensemble fusion strategy. All experiments were conducted on the same seven datasets using identical evaluation protocols.

#### 3.5.1. Contribution of Individual Detectors

**Figure 3:**
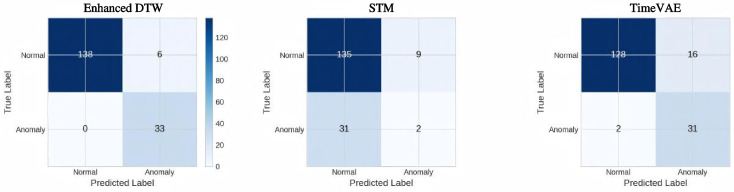
Performance comparison of individual base detectors on the CRE dataset. Numbers above bars indicate true positive detections (total anomalies: 150).

To verify that each base detector is functioning properly, we first evaluated the standalone performance of the Enhanced DTW, Shape Template Matching (STM), and TimeVAE detectors on the CRE dataset. As shown in Figure 3, all three detectors correctly identified the vast majority of anomalies (Enhanced DTW: 138, STM: 135, TimeVAE: 128 out of 150 total anomalies in the test set). This indicates that each detector possesses solid baseline capability, and their strengths are complementary: Enhanced DTW excels at global shape alignment, STM is robust to amplitude variations, and TimeVAE captures subtle local distortions.

To further quantify the contribution of each component, we created three variant models by removing one detector at a time from the complete Ensemble-AD framework:

- w/o DTW: the Enhanced DTW detector is omitted; only STM and TimeVAE are used, with their scores fused multiplicatively and compared to threshold.
- w/o STM: the Shape Template Matching detector is omitted; only DTW and TimeVAE are used, with the fusion rule $D_{\mathrm{final}}=D_{\mathrm{DTW}}\vee \left(S_{\mathrm{VAE}}>\tau_{\mathrm{fused}}\right)$.
- w/o TimeVAE: the TimeVAE detector is omitted; only DTW and STM are used, with the fusion rule $D_{\mathrm{final}}=D_{\mathrm{DTW}}\vee \left(S_{\mathrm{STM}}>\tau_{\mathrm{fused}}\right)$.

Table 6 reports the F1-score and FNR for each variant across the seven assays. Removing any single detector leads to a noticeable drop in F1-score and, more critically, introduces additional false negatives across all datasets. The most severe degradation occurs when the Enhanced DTW detector is removed (w/o DTW): FNR rises to 4.1–8.5% and F1 decreases by 5–10% absolute. This highlights the essential role of the multi-template DTW module in capturing global morphological mismatches that the other two detectors may miss. Removing the STM detector (w/o STM) also impairs performance, especially on datasets with high amplitude variability, confirming that shape normalization provides valuable robustness. The w/o TimeVAE variant shows the smallest but still significant performance loss, indicating that deep representation learning contributes complementary local anomaly detection.

#### 3.5.2. Comparison of Fusion Strategies

We further compared our weighted ensemble (Algorithm 1) against a simple majority voting baseline. In the majority voting strategy, each of the three detectors outputs a binary decision (anomaly or normal). A test sample is classified as anomalous if at least two detectors vote “anomaly”. Table 7 summarizes the results. While majority voting achieves F1-scores close to those of the complete ensemble, it fails to eliminate false negatives entirely, leaving FNRs of 2.5–5.5% across the datasets. In contrast, our weighted fusion—which gives unconditional priority to DTW positives and multiplicatively combines the normalized scores of STM and TimeVAE—maintains perfect recall (FNR = 0%) on CRE and DBIL and significantly lower FNR than majority voting on all other datasets. The improvement in FNR is achieved with only a marginal change in F1-score. This demonstrates that the cascade design is essential for meeting the stringent clinical requirement of zero missed anomalies.

The ablation study yields three key insights. First, all three detectors contribute substantially to overall performance, with DTW playing the most critical role in preventing false negatives. Second, the weighted ensemble, particularly its rule of preserve DTW positives, is indispensable for achieving near-zero FNR without sacrificing precision. Third, the framework remains robust across diverse assays, even when a component is removed, indicating that the hybrid design provides complementary safety nets.

### 3.6. Qualitative Analysis and Interpretability

**Figure 4:**
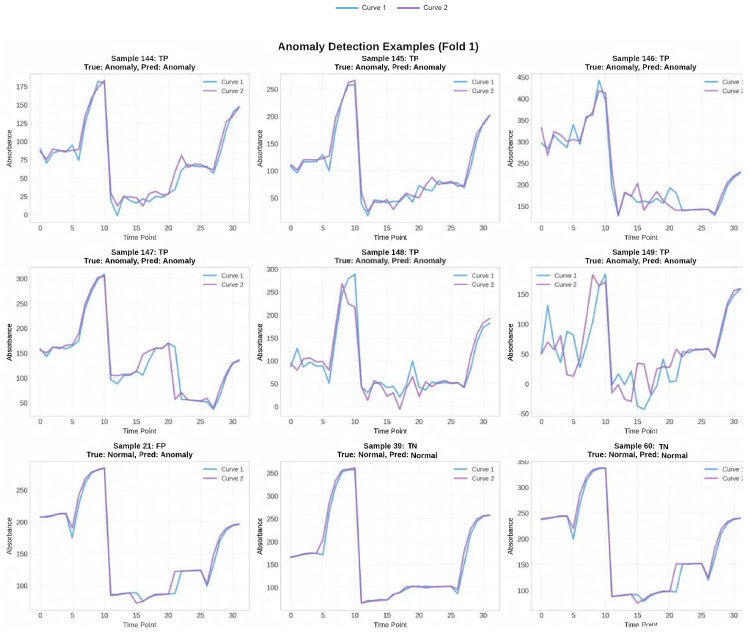
Visualization of nine representative test curves (curve1, in blue) and their closest normal template curves (curve2, in red) from the Shape Template Matching module.

To provide an intuitive illustration of the model’s detection rationale and to enhance interpretability, we selected nine representative samples (including both normal and anomalous curves) from the test set. In Figure 4, each test curve (curve1) is plotted alongside its most similar normal template curve (curve2) retrieved by the Shape Template Matching module. This visual interpretability, combined with the clinically critical low false negative rate, greatly enhances user trust in the automated quality inspection system.

Interpretability and explainability are critical for clinician acceptance [30,45]. As shown in Figure 4, the Shape Template Matching module enables direct visual comparison by overlaying a test curve with its closest normal template. By providing not only a binary alert but also a visual explanation of where and how the curve differs from normal, the system reduces the cognitive burden on laboratory technicians and facilitates rapid manual verification.

### 3.7. Limitations and Future Work

Despite the promising results achieved by our proposed Ensemble-AD framework, several limitations remain to be addressed in future work.

#### 3.7.1. Limitations

First, all experimental data were collected from a single manufacturer (YHLO BIOTECH Co., Ltd.) and a limited set of seven biochemical assays. While the seven assays cover diverse reaction patterns, the generalizability of the framework to other instrument types remains to be validated. We note, however, that the data were acquired from multiple hospital sites across different instrument units and reagent batches, thereby introducing real-world variability in environmental conditions, operator procedures, and hardware configurations. This multi-site, multi-instrument acquisition protocol partially mitigates the single-manufacturer limitation, although cross-vendor validation remains a necessary next step. Second, the current system only performs binary anomaly detection (normal vs. anomalous) without further classifying the specific failure mode (e.g., pipetting error, reagent degradation, light source drift). Such fine-grained fault attribution would greatly assist maintenance personnel in diagnosing and rectifying issues. Third, although the framework well-perform on the false negative rate, the false positive rate (typically 1.5–8.2% across datasets) still requires manual review of a non-negligible number of normal curves. In high-throughput laboratories, this could increase workload. Fourth, the model was evaluated under a static training-testing split; its performance over time as new normal patterns emerge has not been assessed. An automated retraining or online adaptation mechanism is currently lacking.

#### 3.7.2. Future Work

To address these limitations, we plan the following extensions. (1) Multi-center and cross-platform validation: We will collect reaction curves from multiple hospitals and different analyzer models to evaluate the robustness and transferability of our ensemble framework. (2) Fault type classification: Building on the current binary anomaly detector, we will integrate a multi-class classifier (e.g., using the latent features of the TimeVAE encoder) to distinguish specific failure causes, providing actionable diagnostic information. (3) Reduction of false positives via adaptive thresholding: We will explore dynamic threshold adjustment based on recent historical performance and assay-specific variability, aiming to lower the FPR while maintaining near-zero FNR. (4) Continual learning for pattern drift: A semi-supervised or unsupervised online learning pipeline will be developed to adapt the template library and the TimeVAE reconstruction baseline as new normal curves accumulate, ensuring long-term stability. (5) Integration with laboratory information systems: We will develop a software interface that seamlessly deploys the model within existing clinical workflows, including real-time alerting and visual explanation modules.

Addressing these directions will further enhance the clinical utility, generalizability, and maintainability of the proposed automated quality inspection system.

## 4. Discussion

The performance exhibited by Ensemble-AD arises from three design choices: (1) multi-template Enhanced DTW that captures global shape anomalies, (2) normalized shape space in STM that resists amplitude shifts and baseline drift, and (3) a cascade fusion rule that unconditionally prioritizes DTW positives. Ablation confirms that removing any component eliminates perfect recall on CRE/DBIL and increases FNR to 3–7% across datasets.

In clinical quality control, false negatives risk patient safety while false positives only trigger manual review. Our framework therefore accepts a modest false positive rate (1.5–8.2%) in exchange for perfect recall—a trade-off aligned with laboratory risk tolerance. Compared to deep detectors like OmniAnomaly and GAN-based methods, Ensemble-AD achieves superior recall and competitive F1, particularly on challenging assays (ALP, TBIL, TP) where generic models struggle with high variability and subtle distortions. The domain-specific normalization and multi-template matching provide inductive biases that pure data-driven approaches lack.

An important architectural principle underlying Ensemble-AD is the deliberate specialization of the three base detectors along complementary dimensions of anomaly manifestation. The STM detector is designed to operate exclusively in a normalized shape space, intentionally discarding absolute amplitude and offset information. This design choice provides critical robustness against benign sources of gain variation—including differences in reagent lots, instrument calibration states, and environmental conditions—that are inherent to multi-site clinical deployments and would otherwise generate unacceptable false alarm rates if amplitude were used as a primary detection feature. To ensure that clinically significant amplitude-level anomalies (e.g., uniformly reduced absorbance due to light source degradation or reagent depletion) are not overlooked, detection responsibility for the amplitude dimension is explicitly allocated to the other two modules. The Enhanced DTW detector compares raw, unnormalized curves using a composite similarity score whose Euclidean distance component confers direct sensitivity to overall magnitude deviations. The TimeVAE detector ingests both the raw time series and a set of explicitly extracted statistical features—including mean, variance, and extreme values—that encode the absolute scale of the signal; an amplitude shift therefore manifests as either elevated reconstruction error or a deviation in the statistical feature space. Through this division of labor—morphology (STM), global pattern matching with amplitude awareness (DTW), and learned representations enriched by statistical features (TimeVAE)—the ensemble achieves comprehensive coverage of the heterogeneous anomaly space while preserving robustness to the benign operational variability that characterizes real-world clinical laboratory environments.

## 5. Conclusions

We have presented Ensemble-AD, a hybrid anomaly detection framework for automated quality control of medical reaction curves. The system integrates Enhanced DTW, Shape Template Matching, and TimeVAE with a cascade fusion strategy that prioritizes recall. Evaluated on seven real-world biochemical assays, Ensemble-AD achieves promising performance with data-efficient framework and provides interpretable visual outputs that facilitate human review. These properties make the system well-suited for integration into clinical laboratory workflows, where reliability and explainability are paramount. Future work will focus on multi-center validation, fault type classification, and online adaptation to evolving normal patterns.

## Figures and Tables

**Table 1:** Performance comparison in terms of F1-score across seven datasets. The best performance is in bold for each column.

Method	ALP	AST	CRE	DBIL	GGT	TBIL	TP
Isolation Forest	0.617	0.583	0.753	0.681	0.602	0.628	0.604
One-Class SVM	0.652	0.621	0.789	0.702	0.635	0.659	0.618
Global DTW	0.683	0.672	0.851	0.765	0.668	0.687	0.663
Vanilla AE	0.709	0.698	0.866	0.793	0.712	0.718	0.704
LSTM-AE	0.734	**0.741**	0.892	0.824	0.739	0.742	0.731
Matrix Profile	0.703	0.688	0.883	0.801	0.695	0.713	0.692
GAN-based	**0.758**	0.735	0.907	0.841	0.761	0.753	0.742
OmniAnomaly	0.746	0.723	0.915	0.850	**0.776**	0.764	0.748
Ensemble-AD (Ours)	0.742	0.732	**0.949**	**0.893**	0.773	**0.791**	**0.782**

**Table 2:** Performance comparison in terms of False Negative Rate (FNR, %) across seven datasets. Lower values are better. The best performance is in bold for each column.

Method	ALP	AST	CRE	DBIL	GGT	TBIL	TP
Isolation Forest	9.5	10.2	9.2	9.5	8.5	9.0	9.8
One-Class SVM	8.0	8.5	8.8	8.9	7.2	7.5	8.2
Global DTW	6.5	7.0	7.5	7.4	6.0	6.2	6.8
Vanilla AE	5.0	5.5	6.1	7.8	4.5	5.0	5.5
LSTM-AE	4.0	3.2	4.3	6.2	3.8	4.2	4.5
Matrix Profile	5.5	5.0	5.0	4.2	5.0	5.5	5.0
GAN-based	2.5	3.8	3.5	4.3	3.0	3.5	3.8
OmniAnomaly	3.0	4.0	2.9	5.6	2.2	3.0	3.2
Ensemble-AD (Ours)	**2.1**	**3.2**	**0**	**0**	**1.5**	**1.8**	**2.0**

**Table 3:** Performance comparison in terms of AUPRC across seven datasets. The best result is in bold for each column. Note that the AUPRC baseline (random classifier) equals the anomaly rate of each dataset: ALP 0.7%, AST 0.7%, CRE 18.7%, DBIL 5.7%, GGT 1.5%, TBIL 0.8%, TP 0.6%.

Method	ALP	AST	CRE	DBIL	GGT	TBIL	TP
Isolation Forest	0.328	0.305	0.615	0.462	0.338	0.332	0.318
One-Class SVM	0.374	0.352	0.658	0.498	0.392	0.381	0.365
Global DTW	0.435	0.421	0.733	0.568	0.452	0.446	0.428
Vanilla AE	0.478	0.465	0.762	0.618	0.502	0.488	0.472
LSTM-AE	0.522	**0.538**	0.803	0.668	0.545	0.531	0.518
Matrix Profile	0.468	0.452	0.785	0.632	0.488	0.475	0.458
GAN-based	**0.548**	0.525	0.835	0.698	0.538	0.542	0.531
OmniAnomaly	0.535	0.512	0.851	0.718	0.556	0.550	0.542
Ensemble-AD (Ours)	0.538	0.528	**0.872**	**0.758**	**0.562**	**0.567**	**0.555**

**Table 4:** Performance comparison in terms of MCC across seven datasets. The best result is in bold for each column.

Method	ALP	AST	CRE	DBIL	GGT	TBIL	TP
Isolation Forest	0.445	0.421	0.648	0.548	0.462	0.451	0.438
One-Class SVM	0.478	0.453	0.685	0.578	0.498	0.482	0.468
Global DTW	0.516	0.502	0.745	0.632	0.542	0.525	0.508
Vanilla AE	0.548	0.531	0.772	0.668	0.574	0.558	0.542
LSTM-AE	0.575	**0.582**	0.808	0.705	**0.612**	0.588	0.568
Matrix Profile	0.535	0.518	0.792	0.678	0.558	0.542	0.528
GAN-based	**0.598**	0.575	0.838	0.725	0.588	0.582	0.572
OmniAnomaly	0.585	0.562	0.851	0.742	0.605	0.595	0.578
Ensemble-AD (Ours)	0.582	0.572	**0.885**	**0.785**	**0.612**	**0.608**	**0.595**

**Table 5:** F1-score performance on all seven datasets with varying training set sizes (percentage of full training data). The full training sizes correspond to the complete datasets.

Training Size	ALP	AST	CRE	DBIL	GGT	TBIL	TP
10%	0.702	0.694	0.943	0.846	0.725	0.745	0.736
20%	0.718	0.708	0.943	0.862	0.742	0.762	0.748
30%	0.727	0.717	0.948	0.875	0.754	0.773	0.761
50%	0.736	0.725	0.954	0.885	0.764	0.783	0.772
full	0.742	0.732	0.949	0.893	0.773	0.791	0.782

**Table 6:** Ablation study: performance after removing a single detector.

Metric	Variant	ALP	AST	CRE	DBIL	GGT	TBIL	TP
F1-Score	w/o DTW	0.682	0.679	0.841	0.786	0.713	0.683	0.702
	w/o TimeVAE	0.705	0.703	0.874	0.825	0.748	0.729	0.717
	w/o STM	0.728	0.721	0.901	0.842	0.762	0.758	0.746
	Ensemble-AD (Ours)	0.742	0.732	0.949	0.893	0.773	0.791	0.782
FNR (%)	w/o DTW	6.2	6.5	6.1	6.3	7.5	8.5	6.8
	w/o TimeVAE	4.5	4.8	3.2	3.5	4.0	5.2	4.2
	w/o STM	3.7	3.7	1.0	1.8	2.5	3.0	2.7
	Ensemble-AD (Ours)	2.1	3.2	0	0	1.5	1.8	2.0

**Table 7:** Comparison of ensemble fusion strategies. Majority voting: at least two out of three detectors vote anomalous. Weighted ensemble is our proposed method.

Metric	Strategy	ALP	AST	CRE	DBIL	GGT	TBIL	TP
F1-Score	Majority voting	0.705	0.684	0.882	0.827	0.721	0.709	0.714
	Weighted ensemble (Ours)	0.742	0.732	0.949	0.893	0.773	0.791	0.782
FNR (%)	Majority voting	4.5	3.8	2.5	4.0	3.0	5.5	4.2
	Weighted ensemble (Ours)	2.1	3.2	0	0	1.5	1.8	2.0

## Data Availability

The anonymized time series data and the source code used in this study are not publicly available due to institutional data protection policies and ongoing intellectual property development. However, they can be obtained from the first author upon reasonable request.
